# Expression patterns of plexins and neuropilins are consistent with cooperative and separate functions during neural development

**DOI:** 10.1186/1471-213X-6-32

**Published:** 2006-07-17

**Authors:** Olivier Mauti, Rejina Sadhu, Joelle Gemayel, Matthias Gesemann, Esther T Stoeckli

**Affiliations:** 1 Institute of Zoology, University of Zurich, Winterthurerstrasse 190, 8057 Zurich, Switzerland; 2 Brain Research Institute, University of Zurich, Winterthurerstrasse 190, 8057 Zurich, Switzerland

## Abstract

**Background:**

Plexins are a family of transmembrane proteins that were shown to act as receptors for Semaphorins either alone or in a complex together with Neuropilins. Based on structural criteria Plexins were subdivided into 4 classes, A through D. PlexinAs are mainly thought to act as mediators of repulsive signals in cell migration and axon guidance. Their functional role in vertebrates has been studied almost exclusively in the context of Semaphorin signaling, i.e. as co-receptors for class 3 Semaphorins. Much less is known about Plexins of the other three classes. Despite the fact that Plexins are involved in the formation of neuronal circuits, the temporal changes of their expression patterns during development of the nervous system have not been analyzed in detail.

**Results:**

Only seven plexins are found in the chicken genome in contrast to mammals, where nine plexins have been identified. Here, we describe the dynamic expression patterns of all known plexin family members in comparison to the neuropilins in the developing chicken spinal cord.

**Conclusion:**

Our in situ hybridization study revealed that the expression patterns of plexins and neuropilins are only partially overlapping, especially during early and intermediate stages of spinal cord development, supporting both cooperative and separate functions of plexins and neuropilins in neural circuit formation.

## Background

The formation of neuronal circuits crucially depends on the correct navigation of axons to their target areas, where they contact individual target cells to establish synaptic contacts. Axonal navigation is based on sequential growth from choice point to choice point. Pathfinding decisions at choice points and along the axonal trajectory are the consequence of molecular interactions between guidance cues presented by the environment and guidance receptors expressed on the growth cones. A multitude of in vitro and in vivo approaches led to the identification of guidance cues that provide directional information for the navigation of growth cones. The Semaphorins are a structurally diverse family of guidance cues. They are subdivided into eight subfamilies, two found in invertebrates, one in viruses, and five in vertebrates. Initially, Semaphorins were found to be repellents for extending axons. In 1997, Neuropilins were identified as receptors for Semaphorins concurrently in two labs [1-3]; reviewed in [4]. A short time later, a role for Plexins as receptors for Semaphorins was described [5-9]. However, Neuropilins and Plexins had been discovered many years earlier as antigens of monoclonal antibodies raised against proteins from the optic tectum of *Xenopus laevis *[10-12]. In contrast to Neuropilins, which have only been found in vertebrates, Plexins are distributed widely in both vertebrates and invertebrates [13]. The nine Plexins found in vertebrates have been subdivided into four subclasses A-D depending on structural criteria. The largest subfamily is the PlexinA subfamily with four members, followed by the PlexinB subfamily with three members. Subfamilies C and D contain only one member each.

By far the best-studied Plexins are class-A Plexins [14-16]. Their function has been studied predominantly in context of their role as co-receptors (together with Neuropilins) for secreted class-3 Semaphorins [14,16,17]. However, PlexinAs must have functions that are independent of Neuropilins, because they are expressed much more widely in the developing nervous system than Neuropilin-1 and -2. Consistent with this, Plexins were shown to mediate homophilic cell-cell adhesion in a calcium-dependent manner [11]. Furthermore, PlexinAs were shown to mediate effects of membrane-bound class-6 Semaphorins in a Neuropilin-independent manner [18,19].

Until recently, when Sema3E binding to PlexinD1 in a Neuropilin-independent manner was demonstrated during the development of the intersomitic vasculature [20], Neuropilins were thought to be required but not sufficient for class-3 Semaphorin signaling [8,9,21-23]. No signaling component in the cytoplasmic part of Neuropilins could be identified, suggesting that they confer ligand specificity to the complex formed with Plexins, L1, or Off-track [24,25]. In contrast to the secreted class-3 Semaphorins, membrane-associated Semaphorins were shown to bind to Plexins directly [18,19,26,27].

Much less is known about the function of other classes of plexins. An interaction of PlexinB1 with Sema4D has been described, but little is known about the role of PlexinBs in vivo [26,28]. PlexinC1 was demonstrated to interact with Sema7A [7], although the only functional study available to date indicates Integrins rather than PlexinC1 as the function-mediating receptor for Sema7A [29]. PlexinD1 finally was linked to the development of the heart and the vascular system consistent with its predominant expression in endothelial cells [30-32].

As a step toward a better understanding of the diverse roles of Plexins in the developing nervous system, we decided to assess the expression patterns of plexins. Here, we describe the expression of all plexins found in the chicken genome during spinal cord development in comparison with neuropilins.

## Results

### The avian genome lacks homologues for two mammalian plexins

**Figure 1:**
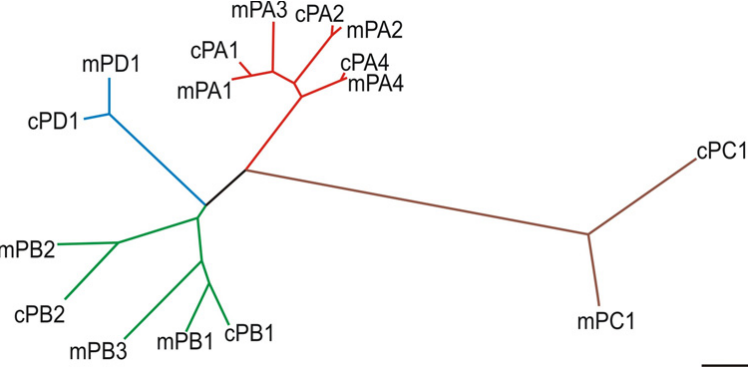
**Phylogenetic tree of the plexin superfamily**. Plexins were aligned on the basis of their sema domain using the CLUSTAL W program. The scale bar represents a substitution rate of 10 amino acids per 100 amino acid residues. For simplicity only mouse (m) and chicken (c) sequences are depicted but the alignment of rat or human sequences gave similar results (data not shown).

In order to identify chicken plexins and neuropilins we performed extensive databank searches using the combined information from the BBSRC ChickEST and the genomic database. These searches indicate that the chicken genome encodes a reduced number of plexins compared to mammals, i.e. only seven instead of nine. While homologous chicken genes for the two unique subfamily members plexinC1 and plexinD1 could readily be identified, fewer chicken plexinAs and plexinBs are present in the chicken genome when compared to mammals (Table 1; Figure 1; [33]). The plexinA subfamily contains no matching chicken sequence for plexinA3. Furthermore, no counterpart for plexinB3 could be extracted from chicken databases (Figure 1 and Table 1). Interestingly, the mouse variants of the two missing plexin genes are located on the X chromosome, implying that a homologous region of this chromosome is absent in chicken. Birds use a Z/W sex determination system instead of the X/Y system found in mammals. Moreover, in contrast to mammals, in chicken the female is the heterogametic sex (ZW), whereas the male is homogametic (ZZ), further suggesting that intense chromosomal rearrangements happened during evolutionary development [34]. These changes are also reflected by the fact that the haploid chicken genome contains 38 autosomes compared to only 19 autosomes in mouse [33,35]. Based on the alignment of human, mouse, and chicken chromosomes, genes found on the mammalian X chromosomes were assigned either to the chicken chromosome 1 or 4 [33].

**Table 1:** Chromosomal localization and EST representation of different plexin genes

plexin	A1	A2	A3	A4	B1	B2	B3	C1	D1
# ESTs	16	23	n.d.	4	4	23	n.d.	6	9
Chick Chr.	12	26	n.d.	1	12	?^1^	n.d.	1	12
Mouse Chr.	6d1	1h6	Xa7.1	6a3.3	9f2	15e3	Xa7.1	10c2	6e3

The number of identified chicken ESTs for each gene and the chromosomal location of the chicken and its corresponding mouse gene are given. Genes that were not detected are abbreviated by 'n.d.' ^1^PlexinB2 has not yet been assigned to a chromosome.

The chicken genome with 1.2 × 10^9 ^bp is only about 40% as long as the human genome, and with an estimated 20'000 to 23'000 genes the chicken contains fewer genes than the mouse or the human genome, although the difference in gene number is not proportional to the reduction in genome size. The chicken genome contains markedly less repetitive sequence and a reduced number of degraded copies of gene sequence, but also fewer duplicated copies of genes overall [33]. Thus, our finding that two plexins are missing is most likely due to their actual absence in the genome rather than our inability to detect them in the databases. Moreover, all attempts to clone the missing chicken homologues using degenerate primers for RT-PCR failed, again implying that the missing genes are indeed not represented in the chicken genome.

Interestingly, while conservation between different chicken plexinAs was in the range of 70 to 90%, depending on the plexin parts used for alignment, conservation between chicken plexinBs was only around 50% (Table 2). This value is not much higher than the values obtained when plexinBs were compared to plexins in other subclasses, suggesting that although placed into the same subclass, plexinB1 and B2 might actually be members of different subclasses.

**Table 2:** Conservation of different domains between Plexin superfamily members

SEMA domain						
plexinA2	57/71					
plexinA4	56/70	59/76				
plexinB1	28/47	28/48	27/47			
plexinB2	28/47	30/49	30/48	36/55		
plexinC1	13/23	12/22	13/22	12/20	12/21	
plexinD1	24/38	23/40	23/40	27/43	24/40	15/28
	plexinA1	plexinA2	plexinA4	plexinB1	plexinB2	plexinC1
PSI domain						
plexinA2	64/73					
plexinA4	74/87	62/77				
plexinB1	50/61	44/55	50/58			
plexinB2	44/65	39/56	39/60	48/66		
plexinC1	42/70	42/76	46/65	48/65	38/57	
plexinD1	37/53	41/61	37/55	40/51	39/53	39/56
	plexinA1	plexinA2	plexinA4	plexinB1	plexinB2	plexinC1

SP domain						
plexinA2	88/95					
plexinA4	82/94	86/95				
plexinB1	56/72	56/73	53/73			
plexinB2	56/74	56/75	56/76	66/79		
plexinC1	49/69	48/68	49/70	45/65	48/66	
plexinD1	56/75	56/74	58/75	55/74	55/74	57/77
	plexinA1	plexinA2	plexinA4	plexinB1	plexinB2	plexinC1

Pairwise alignment of individual domains of chicken Plexin proteins indicate that the intracellular SP (sex-plexin) domain is highly conserved between the different family members, whereas the two extracellular domains, SEMA and PSI (plexin/semaphorin/integrin), show far less conservation. Interestingly the conservation between individual domains of chicken plexinB1 and B2 is only slightly higher than the homology between class A and class B plexins, suggesting that chicken plexinBs are evolutionary quite distinct. Numbers indicate percentage of identical/conserved amino acids.

### The expression patterns of plexins are dynamically regulated during early spinal cord development

**Figure 2:**
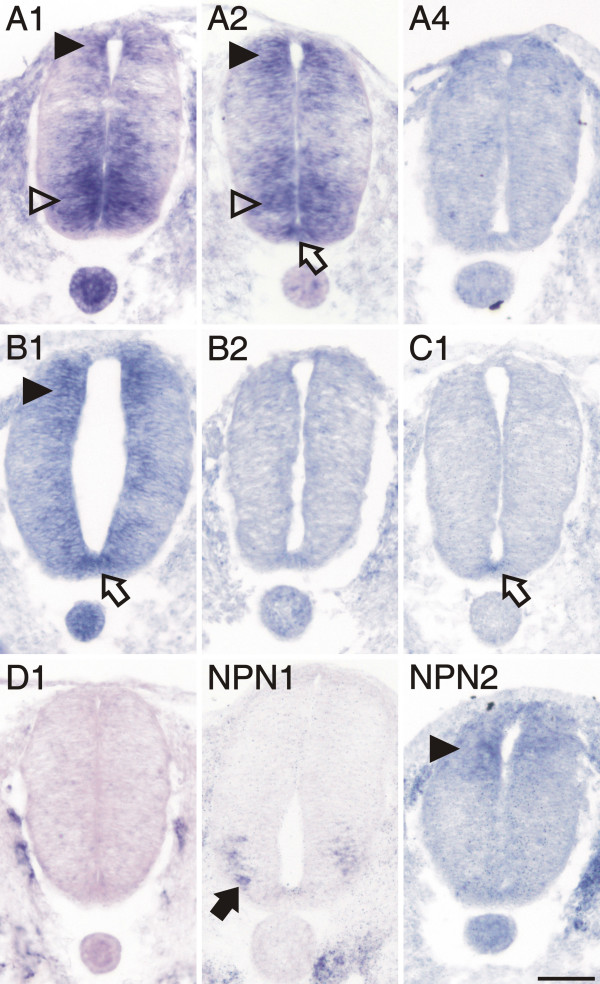
**Expression of plexins and neuropilins in the spinal cord at stage 18 analyzed by in situ hybridization**. PlexinA1, A2, and B1 were detectable with very similar patterns in the dorsal spinal cord at stage 18, the earliest stage we investigated (arrowheads). PlexinA1 and A2 were also expressed in early motor neurons of the ventral spinal cord (open arrowheads). PlexinA2, B1 and C1 were the only plexins expressed in the floor plate (open arrow). In contrast to plexinA1 and A2, npn-1 (NP1) was expressed only in motor neurons that had migrated laterally (arrow). Npn-2 (NP2) was expressed at low levels in the dorsal spinal cord similar to plexinA1, A2, and B1 (arrowhead). Adjacent sections hybridized with the respective sense probes are shown in [Additional file 1]. Bar 50 μm.

**Figure 3:**
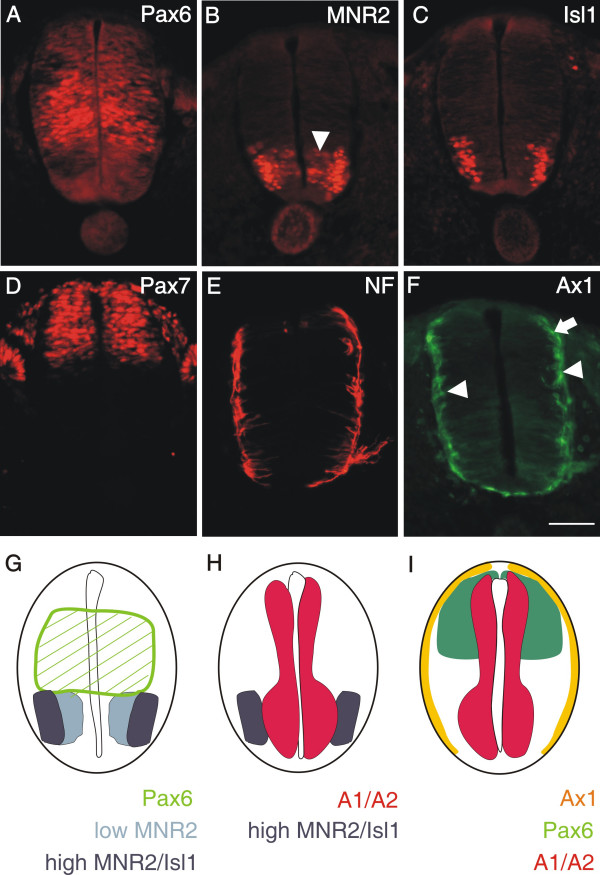
**PlexinA1 and A2 are already expressed in neural precursors**. At stage 18, the homeodomain protein Pax6 labels precursor cells in the intermediate zone of the developing spinal cord (A; [60]). Its ventral expression boundary that is defined by the morphogen Shh released from the floor plate reaches the area with the progenitors of motor neurons expressing MNR2 (B and G). Low levels of MNR2 proteins are seen in progenitor cells located medially (arrowhead in B), where motor neurons are born. MNR2 protein persists and accumulates in postmitotic motor neurons expressing Isl1 (C). In the dorsal spinal cord, Pax7 expression marks a population of precursor cells that give rise to dorsal interneuron subpopulations (D; [37]). Note the decline of Pax7 staining toward the periphery of the spinal cord. Even at stage 19, postmitotic neurons expressing neurofilament proteins are found only at the peripheral margin of the spinal cord (E). At this stage, the dorsolateral population of commissural axons, characterized by the expression of Axonin-1 (arrow in F), starts to extend axons toward the floor plate [38]. At the same, more ventrally located interneurons expressing Axonin-1 also extend axons but their pathway has not been characterized in detail (arrowheads). A comparison of the expression of Pax6 (green hatched area), MNR2 (light and dark blue, characterizing low and high protein levels, respectively) and Isl1 (dark blue) is shown in G. For clarity the mRNA expression domains of plexinA1 and A2 have been added in H (red). Significant overlap was found between the plexin expression domain and early motor neurons characterized by MNR2 staining (H). In the dorsal spinal cord domains of Pax7 (green) and Axonin-1 protein expression (yellow) are compared to the domains expressing plexinA1 and A2 mRNA in I. Plexins do not extend to the periphery of the neural tube where postmitotic neurons expressing neurofilament or axonin-1 are found. Bar in A through F 50 μm.

We started to analyze plexin expression in the lumbosacral spinal cord at stage 18 [36]. At this time, motor neurons are born in the ventral spinal cord and start to extend axons. The first plexins detected in motor neurons were plexinA1 and A2 (Figure 2, compare [Additional file 1] for sense controls). In addition, motor neurons expressed neuropilin-1 (npn-1), but not npn-2. When compared with markers for precursors and mature neurons (Figure 3), respectively, plexinA1 and A2 were clearly expressed already in precursors of motor neurons, whereas npn-1 was restricted to more lateral, mature motor neurons.

**Figure 4:**
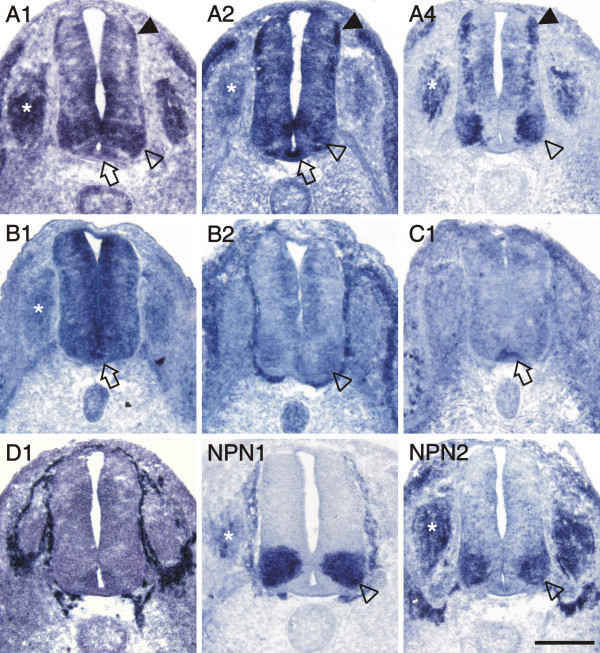
**Both neuropilins and all plexins, except D1, were expressed in the spinal cord at stage 22**. Motor neurons expressed plexinA1, A2, A4, B2, and both neuropilins (open arrowheads). Levels of plexinB2 were very low and plexinA2 appeared to be expressed only in a subset of motor neurons. All class-A plexin were found in dorsal commissural neurons (arrowheads). PlexinA1 and A2, but not A4, were detected in the floor plate (open arrows). Furthermore, the floor plate expressed plexinB1 and C1. PlexinB1 was found throughout the spinal cord with higher levels in the ventricular zone. PlexinD1 was not expressed in the spinal cord at all, but was restricted to endothelial cells and cells ensheathing the spinal cord. The DRGs expressed plexinA1, A2, A4, B1, npn-1 and npn-2 (asterisks). For a comparison sections hybridized with the respective sense probes are shown in [Additional file 2]. Bar 200 μm.

**Figure 5:**
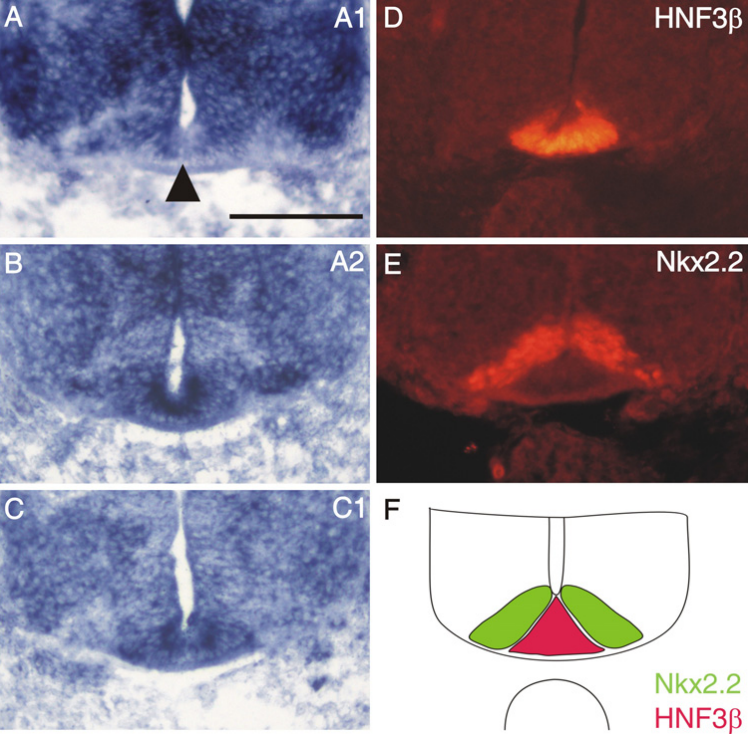
**The expression of plexinA1 in the floor plate is not uniform**. At stage 22, plexinA1 (A) is expressed strongly in lateral floor-plate cells but present only at very much reduced levels or absent altogether in medial floor-plate cells (arrowhead). In contrast to plexinA1, plexinA2 (B) and C1 (C) are found throughout the floor plate at high levels. The floor plate is identified by the expression of the transcription factor HNF3β (D, red in F; [41]). The area exhibiting reduced levels of plexinA1, A2, and C1 adjacent to the floor plate represents area p3, characterized by the expression of the transcription factor Nkx2.2 (E, green in F; [41,50]). Bar 100 μm.

More dorsally, plexinA1, A2, and B1 were expressed in Pax6-positive precursor cells (compare to Figure 3A). In addition, cells in the dorsal spinal cord expressed plexinA1, A2, B1, as well as npn-2 (Figure 2). These cells expressed Pax7 (Figure 3D) but did not express neurofilament proteins (Figure 3E) and therefore represent precursors of dorsal interneurons [37]. At the lumbosacral level of the spinal cord, dorsal commissural neurons derived from these Pax7-positive precursors and characterized by the expression of the cell adhesion molecule Axonin-1 start to extend axons at stage 19 (Figure 3F; [38]). These axons grow toward the floor plate in response to chemoattractants derived from the floor plate, Netrin-1 (reviewed in [39]) and Sonic hedgehog [40]. The majority of these axons have reached the floor plate by stage 22. At this stage, dorsal commissural neurons identified by axonin-1 expression [38,41] expressed all three members of the plexinA class but neither npn-1 nor npn-2 (Figure 4; compare [Additional file 2] for sense controls). Interestingly, plexinA1 and A2 were now also expressed in the floor plate, although plexinA1 was found only in lateral but not medial floor-plate cells (Figure 5). Furthermore, the floor plate was also the earliest site of plexinB1 and C1 expression in the spinal cord (Figure 4 and 5). In contrast to all other plexins, plexinB1 was expressed strongly in the entire ventricular zone and at low levels in almost all cells of the spinal cord at stage 22 and later.

### Motor neurons innervating the hindlimb express a large variety of plexins and neuropilins

**Figure 6:**
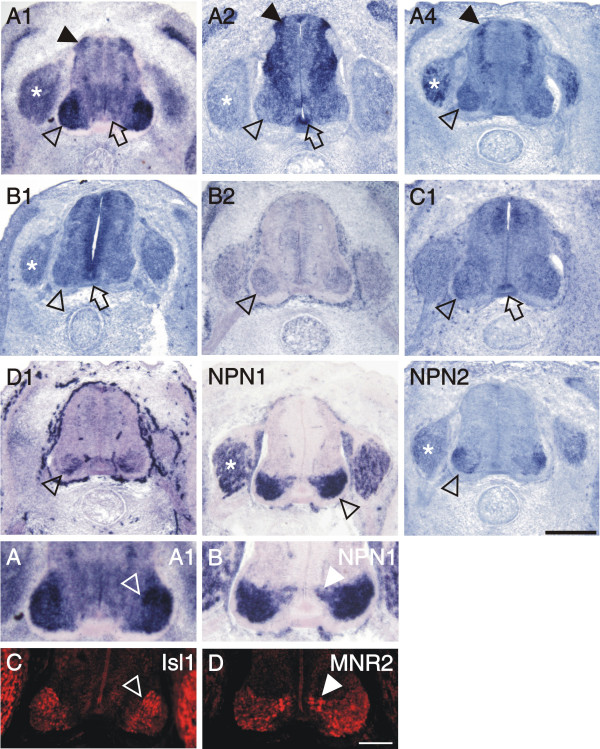
**Stage 25 was the only stage when all plexins and neuropilins were expressed in the spinal cord**. The most prominent mRNA levels were found for plexinAs. They were all detectable in dorsolateral commissural interneurons (arrowheads) and in motor neurons (open arrowheads). Expression of plexinA2 was restricted to some scattered cells in the ventral horn, but absent from lateral areas. Only subpopulations of motor neurons expressed plexinD1, npn-1, and npn-2. PlexinB1, B2, and C1 were found in all motor neurons although at low levels (open arrowheads). Panels A and B are higher magnifications of the sections hybridized with plexinA1 and npn-1, respectively, in comparison to Isl1 (C) and MNR2 (D) staining in adjacent sections. The expression domains of plexinA1, A4, and npn-2 are more similar to the domains expressing Isl1, whereas those for plexinA2, D1, and npn-1 overlap with the more medial motor neurons expressing MNR2. Floor-plate expression was still found for plexinA1 (lateral floor-plate cells only), A2, B1, and C1 (open arrows). The presence of plexinA1, A2, A4, B1, npn-1, and npn-2 in DRGs is indicated by asterisks. Bar 200 μm.

After stage 22, motor axons reach the plexus region where they have to sort out according to their target muscles. While the first decision is primarily a choice to grow either dorsally or ventrally, more refined pathways are chosen at stages 25/26, when individual nerves begin to form [42,43]. At that time, motor neurons can be separated into different subpopulations based on the expression pattern of transcription factors [44-46] or type-II cadherins [47]. At stage 25, all plexins and both neuropilins were expressed in motor neurons (Figure 6). PlexinA1, A4, and, at low levels, B2 were expressed in a pattern overlapping with Isl1-positive motor neurons (Panel C in Figure 6) that coincided with the expression of SC1 a surface marker for motor neurons C1 (not shown). In contrast, the expression of plexinD1 and npn-1 was strong in medial motor neurons labeled by MNR2 but not by Isl1 (Panel D in Figure 6). Npn-2 appeared to be expressed predominantly in a dorsolateral subset of Isl1-positive cells. PlexinA2 and C1 were scattered throughout the ventral horn without clear resemblance to either the Isl1 or the MNR2 pattern and expression levels were rather low. This was also true for plexinB1 that was expressed throughout the neural tube with higher levels found in dorsal commissural neurons, the floor plate, and the ventricular zone. In addition to plexinB1, the floor plate maintained expression of plexinA2, and C1 at stage 25. PlexinA1 was still detectable in lateral floor-plate cells, although at lower levels. Dorsolateral commissural neurons still expressed all plexinAs, plexinB1, and C1, but no neuropilin.

### During intermediate stages of spinal cord development plexin expression is reduced

**Figure 7:**
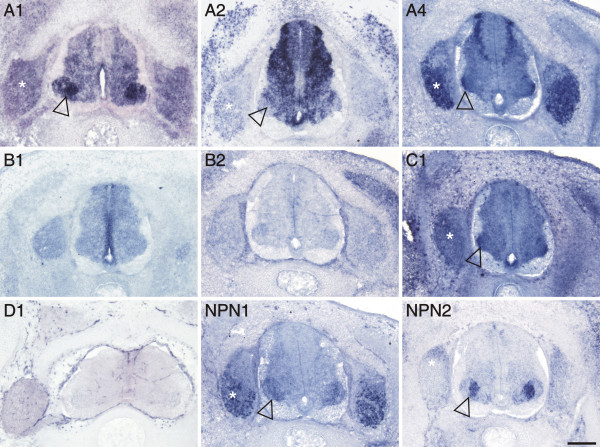
**Expression of plexins and neuropilins in the spinal cord at stage 30**. The transition from early to late phases of spinal cord development was characterized by restriction of plexin-expressing areas. PlexinB2 and D1 were no longer expressed in the spinal cord, the expression levels of plexinB1 had declined considerably compared to stage 25. PlexinA1 was strongly reduced in the dorsal spinal cord, but remained expressed at high levels in motor neurons (open arrowhead). Motor neurons still expressed plexinA2, A4, C1, npn-1 and npn-2 in a subpopulation-specific manner. In DRGs plexinA1, A2, A4, C1, npn-1, and npn-2 were expressed (asterisk). Bar 200 μm.

The early phase of spinal cord development that we analyzed (stages 18 – 25) is characterized by the birth and differentiation of neuronal populations [48-50,37]. This two-day period (E3-E5) revealed fundamental changes in the expression patterns of plexins and neuropilins (compare Figures 2, 4, and 6). In contrast, the expression patterns changed much less during the next two to three days, the intermediate phase of spinal cord development. At stage 30 (Figure 7), the expression pattern of plexinAs remained the same as at stage 25, except for the decrease of plexinA1 in the dorsal half of the spinal cord. PlexinB1 persisted only in the ventral ventricular zone, and plexinB2 disappeared altogether. PlexinC1 was now expressed diffusely throughout the gray matter with higher levels in the lateral motor column and the floor plate. After its transient expression in motor neurons at stages 25 (Figure 6) and 26 (not shown) plexinD1 was no longer expressed in the spinal cord but was restricted to endothelial cells of the blood vessels as described for the mouse [30]. The expression of npn-1 and npn-2 at stage 30 (Figure 7) remained largely complementary in different populations of motor neurons as already indicated at stage 25 (Figure 6). The expression patterns of the neuropilins in motor neurons did not resemble those of any of the plexins. A more detailed analysis of the individual subpopulations expressing plexins and neuropilins would exceed the scope of this study and would require double and triple staining for transcription factors of the ETS and LIM homeodomain families [45,51,52] or comparison with the analysis of type-II cadherin expression patterns in motor neuron pools [47]. The analysis of whole-mount preparations of stage 26 spinal cords did not reveal any substantial changes in the expression patterns of plexins and neuropilins throughout the lumbosacral level [see Additional file 3].

### Late stages of spinal cord development

**Figure 8:**
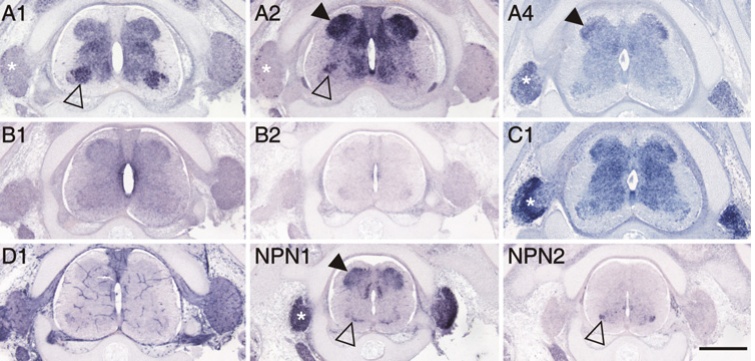
**Expression of plexins and neuropilins in the spinal cord at stage 35**. Plexins became more restricted during late stages of spinal cord development. Motor neurons expressed plexinA1, A2, npn-1, and npn-2 in a subpopulation-specific manner (open arrowhead). PlexinA2 expression levels were very high in the dorsal horn (arrowhead), whereas plexinA4 was found only in a restricted area of the lateral dorsal horn. PlexinC1 was still found throughout the gray matter. In contrast to earlier stages, npn-1 was now expressed in the dorsal spinal cord (arrowhead). Expression of plexinA1, A2, A4, C1, and npn-1 in DRGs is indicated by asterisks. Bar 500 μm.

**Figure 9:**
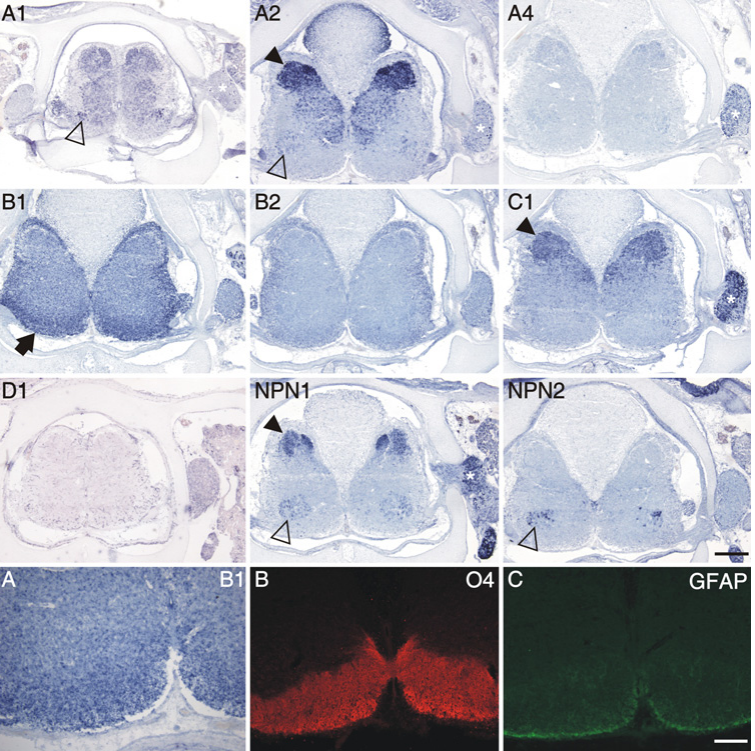
**Expression of plexins and neuropilins in late stages of spinal cord development**. At stage 40, the latest stage we analyzed, plexin expression still changed. The most prominent change was found for plexinB1, which was now restricted to the white matter (arrow; high magnification shown in A). Strong expression in the dorsal spinal cord was still found for plexinA2, C1, and npn-1 (arrowhead). Few positive motor neurons were still found for plexinA1, A2, npn-1, and npn-2 (open arrowheads). DRGs maintained expression of plexinA4, C1, and npn-1 (asterisk). Single scattered cells were still expressing plexinA1 and A2. Panels A – C show high magnifications of a section processed by in situ hybridization to detect plexinB1 (A). Adjacent sections were stained with antibodies recognizing the oligodendrocyte marker O4 (B) and the astrocyte marker GFAP (C). Bar 500 μm for in situ hybridizations of plexins and neuropilins and 200 μm for panels A-C.

During late stages of spinal cord development, between stages 35 (Figure 8) and 40 (Figure 9), motor axons have reached their target muscles and sensory afferents terminate in their specific target layers of the gray matter. Expression patterns of plexins and neuropilins still changed. Overall their levels decreased and their spatial extent became more restricted. Fundamental changes in expression were observed for npn-1 that was found in the dorsal spinal cord during late stages of spinal cord development, but not during early and intermediate stages, where it had been restricted to the ventral spinal cord.

Another significant change was found for plexinB1 that was detected in cells located in the funiculi all around the spinal cord at stage 40 (Figure 9). The expression of plexinB1 (Figure 9A for higher magnification) correlated with areas of O4 expression, a marker for oligodendrocytes (Figure 9B) but not with GFAP expression, a marker for astrocytes (Figure 9C).

At late stages of development, the expression of plexins was restricted to the dorsal gray matter, or was turned off altogether (plexinA4, B2). Strong expression only persisted for plexinA2 and C1 in the dorsal horn, in laminae I – III (Figure 9; [53]).

### DRGs exhibit a dynamic plexin expression pattern

Dorsal root ganglia expressed a variety of plexins during different stages of development. During early stages (stages 22–25), when sensory neurons extend processes into the periphery but before collaterals of central processes are formed ([54], and references therein), plexinA1, A4, and npn-2 transcripts were found at high levels; low levels were detectable for plexinA2, B1, and npn-1 at stage 22 (Figure 4). At stage 25, expression patterns were unchanged for plexins but levels had switched for neuropilins, i.e. npn-1 was expressed at higher, npn-2 at lower levels at stage 25 compared to stage 22 (Figure 6, compare to Figure 4).

At stage 30, when sensory neurons start to extend collaterals into the gray matter of the spinal cord (see [54] and references therein), high levels of plexinA4 were maintained in DRGs (Figure 7), whereas levels of plexinA1 and B1 had decreased. PlexinC1 expression levels started to increase (compare Figures 6, 7, and 8). PlexinA2 remained restricted to a subset of cells. Interestingly, the expression of npn-2 became restricted to sensory neurons located in the dorsal-most part of the DRG at stage 30. As nociceptive, mechanoreceptive, and proprioceptive neurons are segregated in the chicken DRG, these cells are most likely nociceptive neurons [55,53].

At stage 35, plexinA1 and A2 were only found in a few cells located in the periphery of the DRG, where proprioceptive neurons are located (Figure 8; [47,53,55]).

## Discussion

### PlexinAs have a more widespread distribution compared to neuropilins

The distribution of class-A plexins has been studied in more detail than other plexin subfamilies [15,56,57]. Their function has been analyzed predominantly in context of their role as co-receptors (together with Neuropilins) for secreted class-3 Semaphorins [9,56,58,59] but see [18,19,27]. However, based on their much more dynamic and more widespread expression, a Neuropilin-independent role of PlexinAs is evident. In particular, the observation that plexins are expressed in the floor plate is rather unexpected, as the floor plate is the intermediate target of commissural axons, and therefore, the site where ligands for axonal guidance receptors should be expressed. A receptor function of Plexins on floor-plate cells at stage 22 is not obvious, as floor-plate cells do not migrate or undergo structural remodeling at this time. Floor-plate development is terminated much earlier [41,49,50,60,61].

As mentioned above, the chicken genome contains only three members of the A class: plexinA1, A2, and A4. The available expression patterns of class-A plexins [15,56,58,62] fail to reflect the dynamics of plexinA-expression changes shown in this study. At E12.5, plexinA1 is reported to be barely detectable in the mouse spinal cord and DRGs [56,58]. PlexinA2 was found in the roof plate and in interneurons of the ventral spinal cord but not in motor neurons of the trunk region [58]. At more rostral levels, plexinA2 was found in the dorsal spinal cord and in DRGs. Reports of plexinA3 expression were contradictory. Brown et al. [58] reported plexinA3 expression in the ventricular zone and in the floor plate, whereas Cheng et al. [56] reported plexinA3 to be expressed throughout the spinal cord (except for the ventricular zone). The reason for this discrepancy is unclear. Compared to the expression pattern in chick, the pattern of plexinA3 reported by Brown et al. [58] is almost identical to the one we found for plexinB1 at stage 25 (Figure 6). The pattern of plexinA4 in the E12.5 spinal cord of the mouse was restricted to a single population of cells in the lateral spinal cord and to the DRGs [56,62]. With respect to the development of sensory afferents to the spinal cord, E12.5 in the mouse is comparable to stage 30 in the chick. The development of motor neurons at E12.5 is closer to stage 25. We therefore used both stage 25 and stage 30 spinal cords to compare the plexinA patterns between mouse and chick. While plexinA2 and A4 patterns did not change considerably between stages 25 and 30, plexinA1 levels decreased strongly in the dorsal spinal cord and in the DRGs. Consistent with the pattern reported by Brown et al. [58] in mouse, plexinA2 was expressed in the roof plate at stage 25. However, in contrast to the chick, mouse plexinA2 was not expressed in the floor plate.

### PlexinBs are only transiently expressed in neurons

The expression of class-B plexins is difficult to link to any specific function. They are expressed in neurons, although only transiently, and in glial cells. The most prominent and longest lasting expression for a class-B plexin is seen in the ventricular zone, where plexinB1 is expressed from stage 22 to stage 35. This pattern is consistent with the mouse, where plexinB1 was found in the ventricular zone of the spinal cord at E13.5 [63]. At stage 40 in the chick, plexinB1 is restricted to cells in the white matter, presumably oligodendrocytes. This is in contrast to the expression of plexinB1 in mouse [63]. In mouse, plexinB3 is expressed in cells of the white matter resembling the expression of plexinB1 in chick. Furthermore, the expression of plexinB2, which has been reported to match the expression of plexinB1 in the ventricular zone of E13.5 mice [63], differs in chick, where levels of plexinB2 are generally low in the spinal cord and peripheral ganglia. A very weak transient expression of plexinB1 and B2 is detectable throughout the gray matter and in motor neurons between stages 22 and 25, respectively. During that time expression is also found in the DRGs and along the ventral roots, which would indicate that these cells are early Schwann cells aligning with motor axons. In contrast to observations in the mouse [63] plexinBs are not expressed during the time when sensory afferents target their specific layers in gray matter, as no expression is detectable between stages 30 to 35. In the chick, collaterals of primary sensory axons do not form before stage 29 [54]. Thus, a contribution of both plexinB1 and B2 to the formation of central sensory connections seems rather unlikely in the chick in contrast to mouse, where plexinB1 was found in DRGs from E13.5 to E15.5 [63].

### PlexinC1 is not expressed in early stages of neural development

In contrast to plexinAs, which are expressed during the time when neurons extend their axons, plexinC1 is expressed only during late stages of neural development. Neither commissural neurons nor motor neurons express plexinC1 during the time when they approach their first intermediate target, the floor plate and the plexus region, respectively. Interestingly, strong expression of plexinC1 is seen in the floor plate at stage 22 (Figures 4 and 5C), i.e. when the majority of the axons from dorsolateral commissural neurons are in the floor plate [38]. The expression in the floor plate persists through stage 25, when commissural axons have crossed the midline and turned into the longitudinal axis [41]. A massive increase in plexinC1 expression in DRGs is found at stage 35, a time when all other plexins are downregulated compared to their earlier expression levels in DRGs. PlexinC1 expression in DRGs persists at stage 40. Thus, plexinC1 starts to be expressed in all neuronal subpopulations that we analyzed when axons have completed the navigation to their targets suggesting that plexinC1 might be involved in target recognition rather than pathfinding. This would be consistent with the finding of Pasterkamp et al. [29] who reported that the effect of Semaphorin7A, which binds to PlexinC1 with high affinity [7], on axon growth was independent of PlexinC1 but rather mediated by Semaphorin7A's interaction with Integrins.

### PlexinD1 is expressed predominantly in endothelial cells

PlexinD1 is expressed transiently in motor neurons in a subpopulation-specific manner between stages 24 and 26. It is not expressed in the spinal cord during initial axon growth of motor neurons. Only after motor axons have reached the plexus region, some medially located cell populations express plexinD1 at stage 25. As described in mouse [30,31], plexinD1 is predominantly expressed in endothelial cells of the developing intersomitic blood vessels during early stages of development and in blood vessels in general during later stages.

### The expression of neuropilin-2 in chick is much more restricted than in mouse

The expression of npn-2, but not npn-1, in the developing chicken spinal cord differs considerably from the expression pattern reported in mouse. Neuropilin-1 is expressed in DRGs and motor neurons in E10.5 and E12.5 mouse spinal cord [1,58] in a pattern that is similar to the expression in chick at stages 22–25 (Figures 4 and 6). The expression seen in mouse spinal cord at E13.5 [1] is virtually the same as seen in chick at stage 35 (Figure 8).

In contrast to the widespread expression of npn-2 in the mouse spinal cord between E10.5 and 12.5 [1,58,64,65], the expression in the embryonic chicken spinal cord is much more restricted at comparable stages. In mouse, npn-2 is expressed very strongly in dorsal commissural neurons, in ventral populations of interneurons, in motor neurons, and in the floor plate. Functional studies have identified a role for npn-2 in commissural axon pathfinding in the mouse [66]. In chick, npn-2 is not expressed in the dorsal spinal cord at comparable stages. It is restricted to some pools of motor neurons and becomes further restricted with increasing embryonic age. Moreover, npn-2 is never expressed in the embryonic chicken floor plate.

## Conclusion

Our analysis of plexin and neuropilin expression during development of the spinal cord reflects their dynamic regulation that coincides with time windows of axon growth and guidance of different neuronal populations. PlexinAs are expressed more widely than any other class of plexins both temporally and spatially. Notably they are also expressed more widely than the neuropilins, indicating that they must have functions that are Neuropilin-independent. This aspect is underestimated when global expression patterns are compared rather than the detailed subpopulation-specific expression patterns. PlexinBs are expressed during early and intermediate stages of spinal cord development but always at low levels and rather ubiquitously. PlexinC1 is expressed predominantly during late stages of development, whereas plexinD1 is expressed only very transiently in the spinal cord.

## Methods

### Assembly of chicken plexin cDNAs

cDNA sequences for chicken plexins were assembled using the combined information from the ChickEST [67] and the chicken genomic database [68]. Seven different genomic regions coding for plexins were identified using the tblastx alignment algorithm on available vertebrate plexins. The corresponding genomic fragments were downloaded and analyzed using the Genscan gene prediction program [69]. Putative cDNA and protein sequences were compared to the corresponding mammalian homologues and Genscan prediction errors were corrected by manual inspection of the intron/exon boundaries. Gaps in the assembled sequences, due to inaccurate or incomplete genome sequencing, were filled by corresponding EST sequences where possible. A total of 85 chicken ESTs covering parts of 7 different plexins were identified. Among these, 65 contained parts of the coding sequence, whereas the rest covered only parts of the UTR. Sequence alignment of genomic and EST sequences was done using the SeqMan software (Lasergene, DNASTAR). Based on overlapping EST sequences that were supplemented with genomic sequences, 3'UTR sequences were added to the coding sequence. UTR sequences were terminated at the first polyadenylation AATAAA/ATTAAA sequence that followed after verified chicken UTR EST sequences. Using this combined approach a total of 5 complete and 2 partial cDNA sequences for plexins could be assembled.

### Phylogenetic tree assembly and analysis of domain identity

The domain structure of representative members of the chicken and mouse plexin superfamily was obtained using the SMART program [70]. Individual domains were extracted from the sequence using predicted domain boundaries. Conserved domains from the different plexin subfamilies were aligned using the CLUSTAL W alignment algorithm [71,72] provided by the MegAlign software (Lasergene, DNASTAR). Obvious mistakes in domain boundary prediction were manually adjusted. For better representation alignment files were exported into the TREEVIEW software, enabling the graphical representation of the unrooted tree [73]. Identical and conserved amino acids within individual domains were determined by pairwise alignment using the bl2seq program [74].

### Preparation of in situ probes

The chicken cDNA plasmids derived from the ESTs ChEST53D13 (plexinA1), ChEST128L21 (plexinA2), ChEST1014M19 (plexinA4), ChEST890P9 (plexinB1), ChEST799I19 (plexinB2), ChEST860K1 (plexinC1), ChEST867E24 (plexinD1), ChEST110K21 (npn-1), and ChEST675H12 (npn-2) were linearized using restriction endonucleases NotI or EcoRI (Roche). The linearized plasmids were used as templates to produce DIG-labeled in situ probes. For this purpose 1 μg of each cDNA was mixed with 3 μl DIG RNA labeling mix (Roche; 10 mM NTP). Three μl T3 or T7 RNA polymerase (Promega, 20 U/μl), 6 μl of 5× transcription buffer (Roche), 0.8 μl RNasin (Promega, 40 U/μl), 3 μl 100 mM DTT and DEPC-treated H_2_O were added to a final volume of 30 μl. After incubation at 37°C for 2 hours, 3 μl RNase-free DNaseI (Promega, 1 U/μl) were added to the mix, and incubated at 37°C for 30 minutes. Nuclease treatment was stopped by the addition of 2 μl 0.5 M EDTA (pH 8.0). The cRNA probes were LiCl-precipitated and dissolved in 50 μl of DEPC-treated H_2_O.

### In situ hybridization

Chicken embryos were collected at indicated embryonic stages, fixed for 2 hours in 4% paraformaldehyde (PFA) and cryoprotected with 25 % sucrose in 0.1 M sodium phosphate buffer overnight. The embryos were embedded in Tissue-Tek O.C.T compound (Sakura), cut transversally into 20–25 μm sections at a temperature of -20°C, and mounted on Superfrost Plus microscope slides (Menzel-Glaeser). After drying the slides for 30 minutes at 37°C, they were stored at -20°C until further use. For all steps until hybridization we used diethyl pyrocarbonate (DEPC) treated H_2_O and stock solutions. Sections were washed in PBS for 5 minutes and postfixed in 4% PFA for 30 minutes. They were washed twice in PBS and once in H_2_O for 5 minutes each. Sections were then acetylated for 10 minutes in 1% triethanolamine containing 0.25% (vol/vol) acetic anhydride. After two washes for 5 minutes each in PBS and a 5-minute wash in 2× SSC (0.3 M NaCl, 0.03 M tri-sodium citrate, pH 7.0), sections were prehybridized for 3 hours with 300 μl prehybridization solution per slide. To avoid evaporation of the solution, slides were covered with parafilm (pretreated with 7% H_2_O_2 _in H_2_O) for prehybridization and subsequent hybridization. Hybridization was for at least 16 hours. The prehybridization solution comprised of 5× Denhardt's, 250 μg/ml yeast tRNA, 500 μg/ml herring sperm DNA, 5× SSC, and 50% formamide. For hybridization, 200–500 ng/ml of each cRNA probe was added to the prehybridization solution, 300 μl placed on each slide and covered with parafilm. Slides were hybridized at 56°C in a humidified chamber containing 50% formamide/5× SSC. After hybridization, sections were washed for 5 minutes in 5× SSC at 56°C, 5 minutes in 2× SSC at 56°C, 5 minutes in 0.2× SSC at 56°C, 20 minutes in 50% formamide/0.2× SSC at 56°C, 5 minutes in 0.2× SSC at room temperature, and finally washed two times in detection buffer (0.1 M Tris-base, 0.15 M NaCl, pH 7.5) for 5 minutes each at room temperature. Sections were then blocked for 1 hour in blocking buffer (3% milkpowder in detection buffer) and incubated for 1 hour in anti-DIG-AP antibodies (Roche) diluted 1:5000 in blocking buffer (300 μl/slide) in a humidified chamber. After two 15-minutes washes in detection buffer, sections were washed for 5 minutes in alkaline phosphatase (AP) buffer (0.1 M Tris-base, 0.1 M NaCl, 50 mM MgCl_2_, pH 9.5). For the color reaction, sections were incubated 12–24 hours in a dark humidified chamber in AP buffer (500 μl/slide) containing 337.5 μg/ml nitroblue tetrazolium (NBT; Roche), 175 μg/ml 5-bromo-4-chloro-3-indoyl phosphate (BCIP; Roche), and 240 μg/ml levamisole (Sigma). The reaction was stopped by washing sections 2 times for 10 minutes each in TE buffer (10 mM Tris-base, 1 mM EDTA, pH 8.0), followed by a short dip in H_2_O. Finally, the sections were coverslipped with an aqueous mounting medium (Celvol). Reaction times were the same for all stages for a given probe, except for plexinA1 and A2. For these two probes reaction times for stage 18 were longer than those for older stages to optimize signal to noise rations. Thus, expression levels at st18 cannot be directly compared to older stages.

### Immunohistochemistry

Antibodies recognizing Pax6, Pax7, MNR2, Isl1, and SC1/Ben were obtained from the Developmental Studies Hybridoma Bank. The monoclonal antibody RMO270, recognizing neurofilament protein was purchased from Zymed, rabbit anti-axonin-1 was described earlier [75]. Secondary antibodies goat anti-mouse Cy3 (Jackson Laboratories) and goat anti-rabbit Alexa488 (Molecular Probes) were used to visualize primary antibody binding sites. Sections were prepared as detailed above. The staining protocol was as detailed earlier [54].

## Authors' contributions

OM carried out all the in situ hybridizations and data analyses, RS contributed to probe preparation, tissue sectioning, and immunohistochemistry. JG and MG carried out the bioinformatics analysis, ES conceived and coordinated the study, and wrote the manuscript. Figures were prepared by OM, MG, and ES. All authors read and approved the final manuscript.

## Supplementary Material

Additional File 1 **Sense controls of stage-18 spinal cord sections**. Adjacent transverse sections of stage-18 spinal cords were hybridized with the respective sense probes for a comparison with the antisense probes shown in Figure 2. Bar 50 μm. Click here for file

Additional File 2 **Sense controls of stage-22 spinal cord sections**. Transverse sections of stage-22 spinal cords adjacent to the ones shown in Figure 4 were hybridized with the respective sense probes as a negative control4. Bar 50 μm. Click here for file

Additional File 3 **The expression patterns of plexins and neuropilins do not change significantly within the lumbosacral region of the spinal cord**. Whole-mount preparations of stage 26 spinal cord were used for in situ hybridization to detect plexin and neuropilin mRNAs. At the resolution of whole-mounts no changes were detectable throughout the lumbosacral region of the spinal cord. The only exception being expression levels of npn-2 mRNA that seemed to decrease in some segments of the lumbosacral spinal cord (arrowhead). Bar 500 μm, 200 μm in A-C. Click here for file
