# Supplementary figures and images for: Expression patterns of plexins and neuropilins are consistent with cooperative and separate functions during neural development
Source: BMC Dev Biol. 2006 Jul 17;6:32. doi: 10.1186/1471-213X-6-32 (PMC1543641; doi:10.1186/1471-213X-6-32)

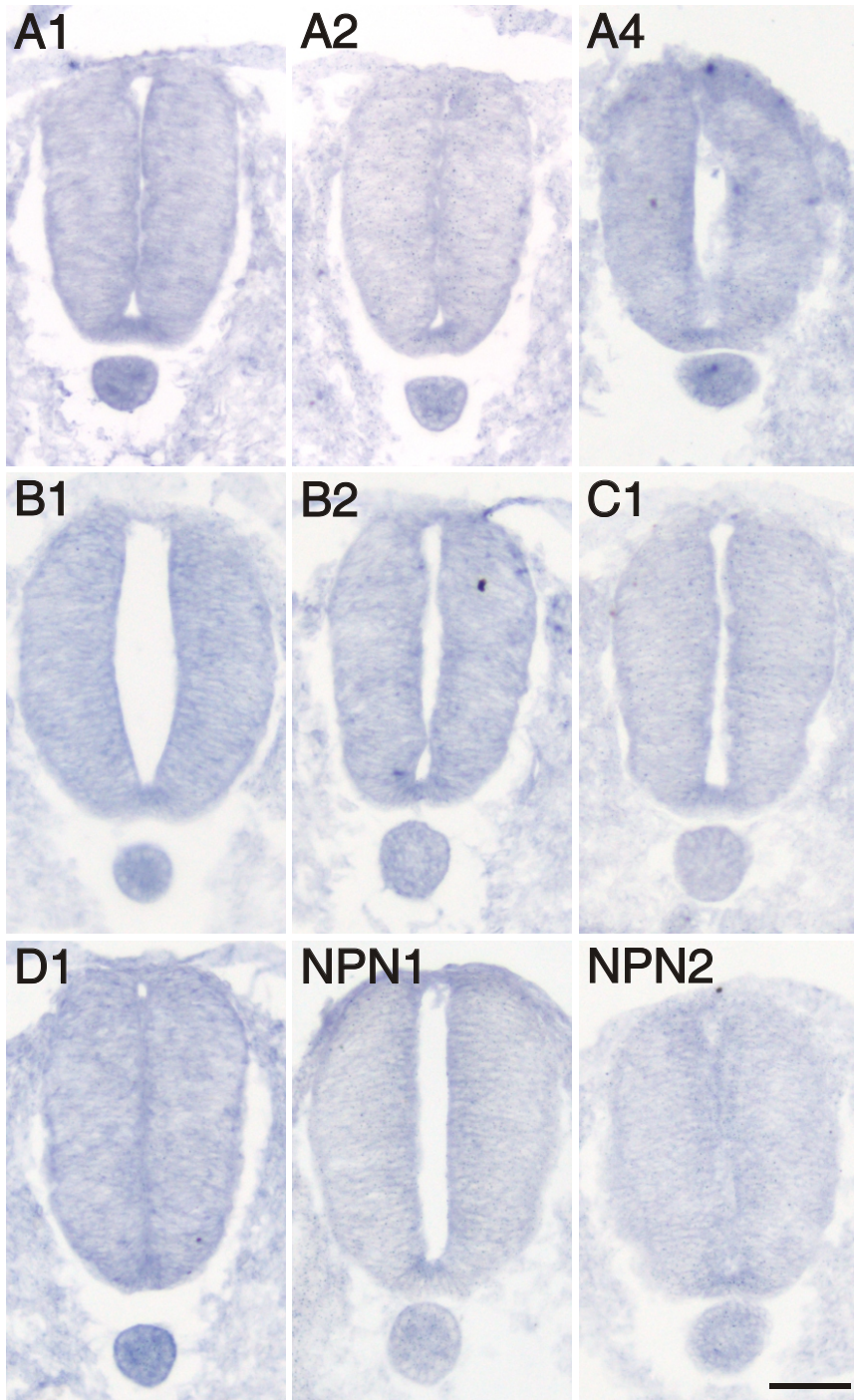

Supplement: Additional File 1 — Sense controls of stage-18 spinal cord sections. Adjacent transverse sections of stage-18 spinal cords were hybridized with the respective sense probes for a comparison with the antisense probes shown in Figure 2. Bar 50 μm. [file 1471-213X-6-32-S1.pdf]

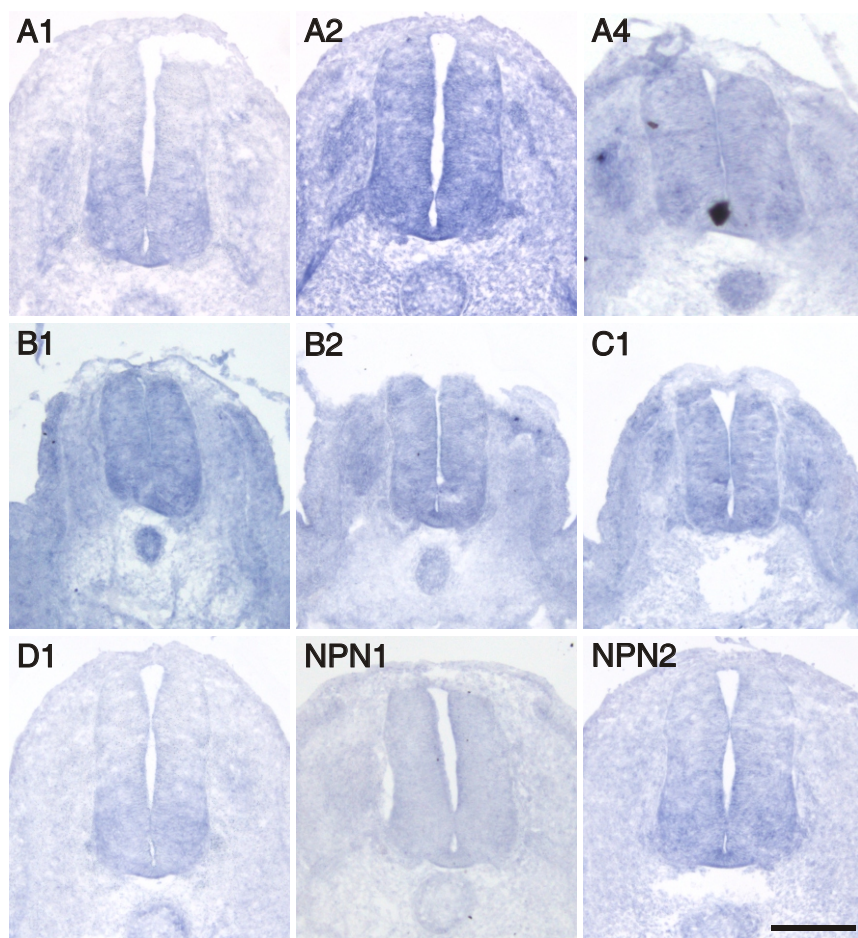

Supplement: Additional File 2 — Sense controls of stage-22 spinal cord sections. Transverse sections of stage-22 spinal cords adjacent to the ones shown in Figure 4 were hybridized with the respective sense probes as a negative control4. Bar 50 μm. [file 1471-213X-6-32-S2.pdf]

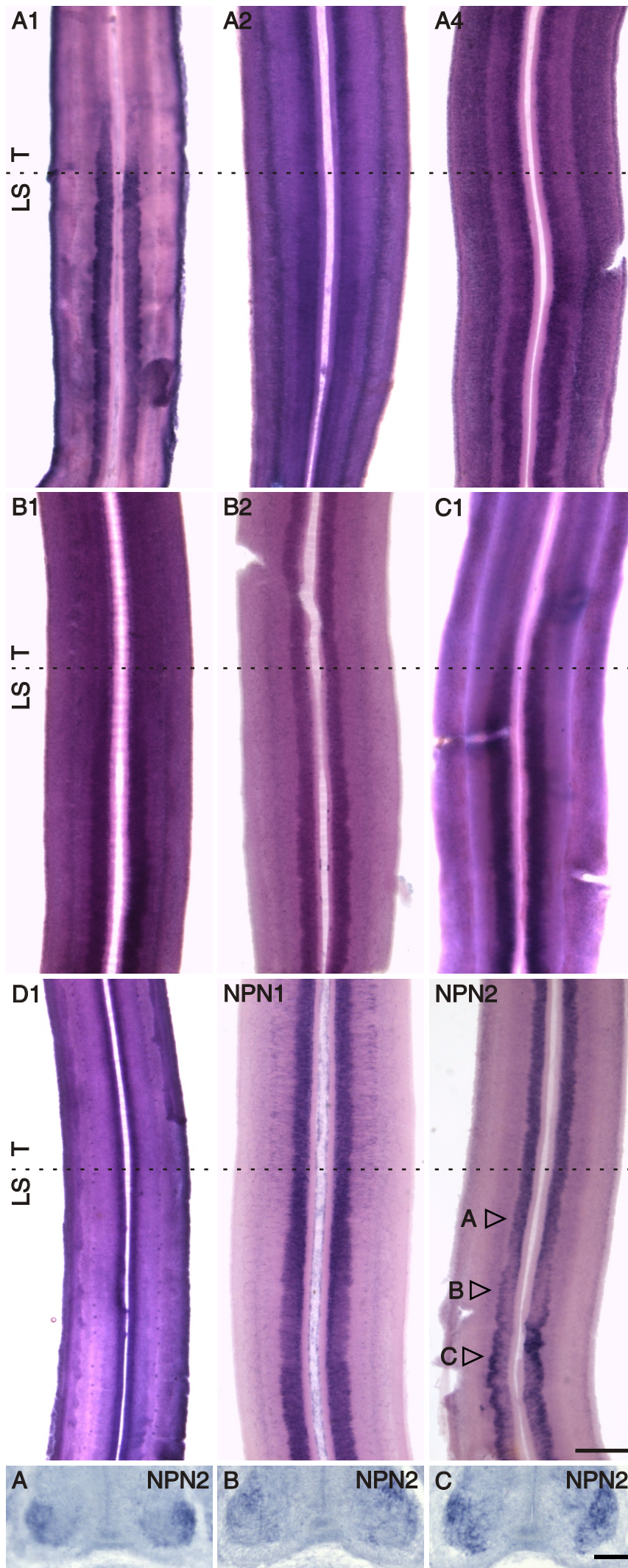

Supplement: Additional File 3 — The expression patterns of plexins and neuropilins do not change significantly within the lumbosacral region of the spinal cord. Whole-mount preparations of stage 26 spinal cord were used for in situ hybridization to detect plexin and neuropilin mRNAs. At the resolution of whole-mounts no changes were detectable throughout the lumbosacral region of the spinal cord. The only exception being expression levels of npn-2 mRNA that seemed to decrease in some segments of the lumbosacral spinal cord (arrowhead). Bar 500 μm, 200 μm in A-C. [file 1471-213X-6-32-S3.pdf]
